# Rural SNAP Participants and Food Insecurity: How Can Communities Leverage Resources to Meet the Growing Food Insecurity Status of Rural and Low-Income Residents?

**DOI:** 10.3390/ijerph17176037

**Published:** 2020-08-19

**Authors:** Emily DeWitt, Rachel Gillespie, Heather Norman-Burgdolf, Kathryn M. Cardarelli, Stacey Slone, Alison Gustafson

**Affiliations:** 1 Family and Consumer Sciences Extension, University of Kentucky, Lexington, KY 40506, USA; rachel.gillespie@uky.edu; 2 Department of Dietetics and Human Nutrition, University of Kentucky, Lexington, KY 40506, USA; heather.norman@uky.edu (H.N.-B.); alison.gustafson@uky.edu (A.G.); 3 College of Public Health, University of Kentucky, Lexington, KY 40506, USA; Kathryn.cardarelli@uky.edu; 4 Department of Statistics, University of Kentucky, Lexington, KY 40506, USA; stacey.slone@uky.edu

**Keywords:** rural, food insecurity, food access

## Abstract

The burden of obesity disproportionately influences poor health outcomes in rural communities in the United States. Various social and environmental factors contribute to inadequate food access and availability in rural areas, influencing dietary intakes and food insecurity rates. This study aims to identify patterns related to food insecurity and fruit and vegetable consumption within a SNAP-eligible and low-income, highly obese rural Appalachian community. A prospective cohort was implemented to identify gaps in resources addressing obesity and food insecurity challenges. SAS 9.4 software was used to examine differences in dietary intakes and shopping practices among SNAP participants. Among participants (*n* = 152), most reported an annual household income less than USD 20,000 (*n* = 90, 60.4%), 29.1% reported food insecurity, and 39.5% reported receiving SNAP benefits within the last month. The overall mean FV intake was 3.46 daily servings (95% CI: 3.06–3.91) among all participants. SNAP participation was associated with food insecurity (*p* = 0.007) and those participating in SNAP were two times more likely to report being food insecure (OR = 2.707, 95% CI: 1.317, 5.563), relative to non-participants. These findings further depict the need for intervention, as the burden of food insecurity persists. Tailoring health-promoting initiatives to consider rurality and SNAP participation is vital for sustainable success among these populations.

## 1. Introduction

The burden of obesity and related chronic diseases disproportionately affects rural communities in the United States (U.S.) more so than their urban counterparts [1]. Theories of social disorganization suggest that the intersection between community structure, such as poverty, socioeconomic status (SES), and residential instability, can result in a void of health promoting culture, infrastructure, and efficacy [2,3]. Previous insights have shown disparaging differences between urban and rural areas on mortality, chronic disease, and screening rates [3,4]. Residents’ limited knowledge of health promoting behaviors may lead to poor health literacy and unhealthy lifestyle behaviors, including poor dietary intakes and sedentary physical activity levels [5,6,7]. Thus, the degree of rurality among geographic areas throughout the U.S. influences the numerous barriers rural communities face and, consequently, their morbidity and mortality rates.

Among rural populations, myriad factors affect obesity rates, though fruit and vegetable (FV) intakes are of great influence and few U.S. adults are meeting recommended amounts [8]. This is particularly true in rural communities, where adults exhibit higher obesity prevalence and are less likely to meet daily FV recommendations due to various social and environmental factors [1,9] relative to their urban counterparts. In addition to individual level factors associated with poor dietary intake, rural residents also face greater rates of food insecurity [10]. A depleted or limited food landscape can predispose residents’ dietary consumption and shopping patterns thereby further influencing their health status, as diet is a contributing factor in several chronic illnesses [11]. While agriculture and food production are prominent in many rural landscapes across the U.S., it is not the case for all rural communities. Rurality does not equate to farmland or local food production, which many would think support food security within these communities. Further, the 2017 Census of Agriculture revealed a decline in number of farms and farmers and in acres of farmland and farmland production [12]. At the local level, there are numerous factors that dictate food production, including geography, terrain, and inadequate resources such as economic hardship or lack of farmers. Those who do operate small farms rely on additional off-farm sources for household income [13]. These factors can also influence the household food environment in rural areas.

Among low income rural populations, the household food environment, including food security and income concerns, are key factors controlling food choice [14]. Rural communities continue to face higher rates of food insecurity, compared with their urban counterparts [10], and food insecurity has been associated with obesity and greater cardiometabolic risk [15]. The Supplemental Nutrition Assistance Program (SNAP) is the largest federally funded nutrition program in the U.S., serving as a household-supporting infrastructure for individuals facing food insecurity. SNAP assists eligible, low-income individuals and families in need throughout the U.S. [16]. While eligibility varies by state, those whose income and resources fall below certain thresholds are able to supplement their food budgets using SNAP benefits [16]. Thus, SNAP is often considered a vital resource for those living in rural communities, as the perpetual SES divide continues between rural and urban settings [17]. At the national level, approximately 16% of those living in rural communities live below the federal poverty line, compared with 12% in urban areas [18]. Due to these income gaps, SNAP participation is higher in rural areas, with 16% of households participating, compared with 13% in urban areas [19]. Additionally, most recent federal data from 2017 indicate that of those eligible for SNAP, participation is higher in rural areas (90%) compared to urban areas (82%), and this participation gap continues to climb [20].

Rural areas account for 63% of counties in the U.S., and 87% of counties with the highest rates of food insecurity [21]. Furthermore, a report from 2018 indicates that 13.3% of those living in rural areas faced food insecurity, compared with 11.5% in urban areas [10]. Resources, such as SNAP benefits, and other programs for those of low SES, are imperative for those in rural communities, as many in these areas are at risk of being food insecure. Thus, initiatives like SNAP can aid in alleviating food insecurity among vulnerable households and improve dietary intakes, when adequate access to nutritious choices are available [22]. Community-based efforts have emphasized the importance of looking at social and physical environments when striving to improve food access [23,24,25]. Therefore, community-based efforts focused on addressing the local food system are necessary to alleviate the barriers related to the procurement of nutritious foods in rural areas. Prioritizing engagement with key stakeholders and community members is vital to consider how to best approach food access initiatives in rural communities.

Conceptually, community-based efforts can be successful in rural communities, as the multifaceted community setting plays a vital role in influencing the food environment and, ultimately, diet choice in these communities. Improving health outcomes pose unique challenges, as resources are sparse and healthcare infrastructure is limited; however, modifying or improving the existing food environment encourages nutritious food choices and shopping behaviors. Nonetheless, environmental triggers and product availability affect the dietary choices individuals make, influencing overall health and obesity status [26]. Given the unique limitations rural communities face, exploring frequented destinations to assess availability can be beneficial to mitigating the barriers that exist [27]. Knowing one’s food environment, SNAP participation, and food insecurity status can influence diet quality, an understanding of the interrelationship among these factors can provide guidance for intervention.

This study aims to identify patterns related to FV consumption and food access within a SNAP-eligible and low-income, highly obese rural Appalachian county in Kentucky. These findings will serve as a baseline to provide context for addressing food insecurity in a remote rural region of the U.S. Baseline findings will guide points of intercept, design future programming to explore the impact rurality has on obesity status, and address the barriers related to accessing nutritious foods within this community and those similar.

## 2. Materials and Methods

The present study is part of a multi-year High Obesity Program (HOP) project through the Centers for Disease Control and Prevention (CDC) to reduce rural obesity prevalence and decrease the risk of chronic disease and preventable mortality. This paper describes one component of the HOP project aimed at providing increased geographic or financial access to nutritious foods. Efforts to improve food access will address food insecurity. This work was completed by leveraging existing Cooperative Extension (CES) infrastructure, with an emphasis placed on community partnership and empowerment, thus enforcing action via established community infrastructure.

The CDC funding announcement identified eligible counties across the U.S. based on their obesity prevalence. The setting for this funded project was one eligible Appalachian county in Kentucky with an adult obesity prevalence greater than 40% per the CDC. The Appalachian region of the U.S. has continued to experience significant decline in life expectancy [28], lack of economic development, and stark out-migration, leaving once fervent and thriving communities destitute, impoverished, and struggling to prosper [29]. This community is reflective of the region, experiencing a persistently high rate of poverty and unemployment, low educational attainment, and food insecurity. The CDC’s Social Vulnerability Index, comprised of social and economic indicators, designates the county as “highly vulnerable.” [30] The county population is approximately 11,200, and declining, with a median household income of USD $35,000 and an estimated 39% of the population living in poverty [31]. The estimated food insecurity rate is 21%, and approximately 31% of households participate in SNAP [32,33].

In order to assure broad community input into all program activities, a health coalition was formed, comprised of key stakeholders including local officials (mayor, magistrates), school representatives (food service director, family resource coordinators), library director, concerned citizens, health department representatives, faith-based organization representatives, and community advocates. The health coalition has been pivotal in establishing partnerships to improve health outcomes within the community. It continues to provide input and direction for all aspects of the current project to identify and implement nutrition-related strategies to address the issue of obesity in the county.

**Figure 1:**
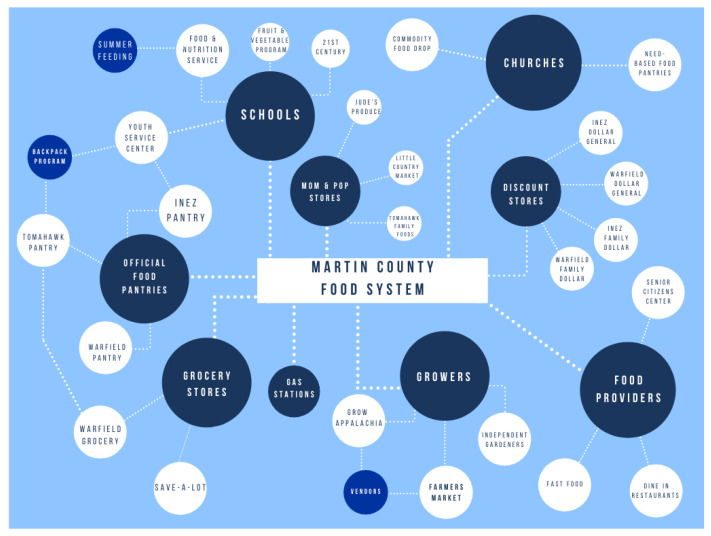
Martin County Food System.

The current study aims to identify gaps in community resources to establish new partnerships that address obesity and food insecurity challenges. Therefore, a formative food system assessment was conducted at baseline to identify potential areas for intervention to enhance healthier food procurement options. Figure 1 outlines the community’s primary food access points identified through the food systems assessment. Findings from the assessment were shared with the coalition to identify potential programmatic efforts to reduce food insecurity within the community.

In alignment with the aim of this study, and to complement community efforts, a prospective cohort was enrolled at baseline for a longitudinal study. The prospective cohort study included a face-to-face survey that occurred in year 1 and will again at years 2 and 3. The University of Kentucky Institutional Review Board (IRB) approved the research, promotional materials, consent forms, and survey instrument.

### 2.1. Baseline Survey Administration

In summer 2019, messages on the county’s CES Facebook page recruited community residents interested in participating in the cohort study. The CES office, a local food pantry, several faith-based organizations, and grocery stores in the county distributed recruitment materials. Furthermore, recruitment occurred through current community programs offered at the CES office. Recruitment messaging continued until enough individuals enrolled to meet the required sample size, which allowed for attrition.

Participants were excluded if they were under 21 years of age, lived outside the county, were non-English speaking, reported plans to move within the next three years, had lived in the county for less than one year, or if they had been diagnosed with cancer. Invited study participants completed the survey via a face-to-face meeting. Prior to survey administration, interviewers verbally reviewed key points of the consent form with participants, who then reviewed the full consent form independently, and provided an opportunity to ask questions or to decline participation. Once deemed eligible, and agreeable to participation, the participant signed the informed consent form and enrolled in the study. A statistical power analysis was performed for sample size estimation and a proposed sample size of 150 adults were recruited to allow for expected attrition.

The prospective cohort study surveys were administered at three locations in the county on various days in fall 2019: the CES office, a local food pantry, and the senior citizens center. The initial date of administration had greater turnout than anticipated by study personnel; several surveys were self-administered as a result (*n* = 24). Moving forward, study personnel modified recruitment processes to schedule appointments for each eligible participant to partake in a verbally administered survey. Those interested contacted study personnel or the county’s CES office to schedule a day and time to participate. This resulted in fewer ineligible participants and complete survey responses. The administered survey took approximately 45–60 min to complete. Participants received a USD $25 incentive to be used a local grocery store as compensation for completing the survey.

**Figure 2:**
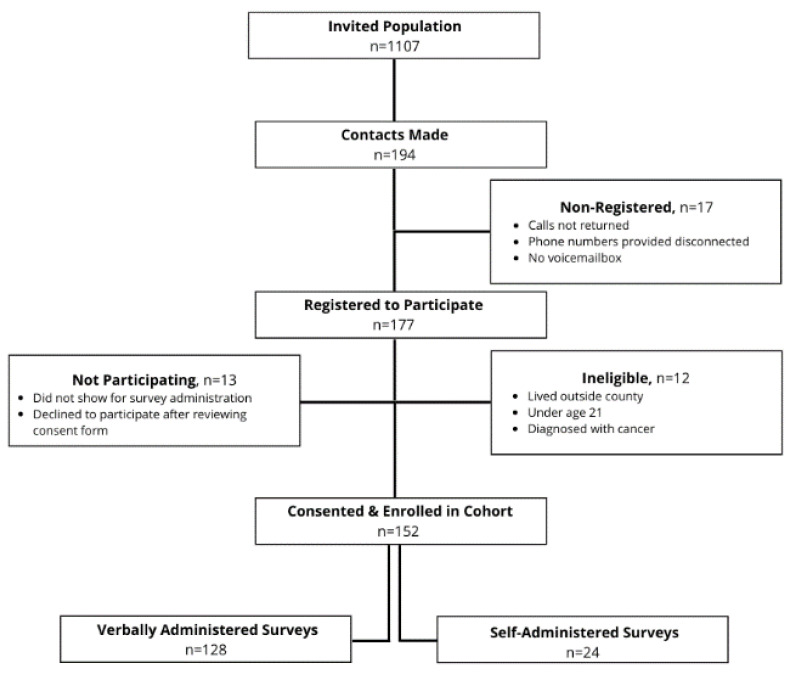
Prospective Cohort Recruitment Processes.

Figure 2 outlines the recruitment and enrollment process for the prospective cohort. The invited population (*n* = 1107) includes all engaged via Facebook and the number of promotional materials distributed through other channels. Of 194 contacts made, 177 individuals registered to participate.

### 2.2. Measures

The survey instrument utilized for this cohort comprised a variety of items to measure FV intake, household environmental measures, food purchasing practices, and demographic characteristics. Demographic items in this analysis included age (in years), gender, preferred language, residential status, highest attained education level, race, and annual household income.

#### 2.2.1. Independent Variables

SNAP participation was assessed by asking: “In the past month, did you or any member of your household receive SNAP benefits or food stamps?” Response options included ‘yes’ or ‘no’.

#### 2.2.2. Dependent Variables

Questions from the National Cancer Institute (NCI) Fruit and Vegetable Intake Screener [34,35] assessed FV intake. The NCI screener asks respondents about usual intake of various FV, ranging from never to ≥5 times per day, and portion sizes for every item (e.g., “Over the last month, how many times per month, week, or day did you eat fruit?” and “Each time you ate fruit, how much did you usually eat?”). Items include 100% fruit juice, fruit, lettuce salad, French fries or fried potatoes, other white potatoes, cooked dried beans, other vegetables, tomato sauce, vegetable soup, and mixtures that included vegetables. Summed items created an overall measure of FV intakes among the sample. This measure served as the primary dependent variable for analysis because increased FV intake is a primary goal of the CDC HOP project.

The secondary dependent variable, food insecurity, was assessed by asking “Which of the following statements best describes the amount of food eaten in your household in the last 30 days?”—enough food to eat, sometimes not enough to eat, or often not enough to eat [36]. “Sometimes not enough to eat” and “Often not enough to eat” were collapsed into “Not enough food to eat” to create a dichotomous assessment of food insecurity.

#### 2.2.3. Covariates

Potential covariates of interest included gender, income, education, and years of residency. To minimize skewedness, income, education level, and residential status categories were collapsed: income was dichotomized as <USD $20,000/year or ≥USD $20,000/year; education categorized as less than high school degree or high school degree or more; and years of residency was dichotomized into <20 years or ≥20 years.

### 2.3. Statistical Analysis

Data were entered into REDCap (Vanderbilt University, Nashville, TN, USA) by trained study personnel and were double-checked to minimize data entry errors. Data were then exported, and analyses were conducted using SAS 9.4 (SAS Institute, Cary, NC, USA). For all analyses, significance was set at *p* < 0.05.

Descriptive statistics, including frequencies and medians, were used to characterize survey responses. The NCI food frequency scores were calculated using the published algorithms [34]. To test comparisons across groups, Wilcoxon rank sum tests were used for continuous measures (age, FV consumption patterns) and Chi-square and Fishers Exact were utilized, as appropriate, for categorical measures. Logistic regression models were fit to assess the predictors of food insecurity. Since FV servings per day were skewed, the data were log transformed and fit with a generalized linear model. Models were adjusted for age, gender, SNAP participation, residency, and income.

## 3. Results

### 3.1. Sample Characteristics

Table 1 details the characteristics of the cohort study population and those of the Martin County population. Of those who registered, there were 152 participants consented and enrolled in the baseline cohort. Participants (*n* = 152) had a mean age of 56 years, with the majority (*n* = 99, 65.1%) identifying as female. Most participants reported an annual household income less than USD $20,000 (*n* = 90, 59.2%) and 39.5% report receiving SNAP benefits within the last month.

### 3.2. Shopping Practices

Table 2 outlines household shopping practices. Respondents shop at grocery stores (80%) or supercenters (20%) and primarily shop there due to price (42%) and location (41%). These practices were not significant when stratified by SNAP participation. Participation in SNAP was associated with an awareness of the farmers’ market (*p* = 0.04), yet not with regularly shopping at this venue (*p* = 0.31).

### 3.3. Fruit and Vegetable Intakes

Findings from the regression analyses are in Table 3. Among all participants, the overall mean FV intake was 3.46 daily servings (95% CI: 3.06–3.91). Males not participating in SNAP (4.45 servings; 95% CI: 3.43–5.76) and females participating in SNAP (3.94 servings; 95% CI: 3.12–4.99) reported highest FV intakes within the sample. There is no significant effect for daily FV intake by residency or education.

### 3.4. SNAP and Food Insecurity

Among all participants, 29.1% reported food insecurity, with a greater percentage of SNAP participants reporting being food insecure (41.7%) than non-participants (20.9%). Logistic regression models indicate that SNAP participation was associated with food insecurity (*p* = 0.007) compared to those not participating in SNAP. Those participating in SNAP were two times more likely to report being food insecure relative to non-participants (OR = 2.707, 95% CI: 1.317, 5.563). When examining food insecurity and dietary intake by SNAP participation stratified by gender (Table 4), the effect of SNAP participation on food insecurity was stronger for females (OR = 3.136, 95% CI: 1.288–7.636), but not significant for males.

## 4. Discussion

It is clear among the literature that numerous factors contribute to high obesity prevalence, including poor nutrition, absence or lack of physical activity practices, and the environment [1,8,9]. Residents of rural communities experience greater obesity prevalence and limited access to healthy choices related to the environments in which they live [9]. These factors shape rural residents’ behaviors, and are well understood as influencing obesity prevalence. Additionally, attention to the food environment is imperative in order to increase consumption of healthier foods within rural communities. Access to healthy foods, such as FV, must be a grounding consideration of obesity prevention efforts within disadvantaged communities such as those found in rural Appalachia.

Rural communities typically face greater financial burden compared with urban communities, particularly in the south, [18] and low household income augments obesity prevalence [37,38]. We did not specifically investigate income within this sample because of collinearity between income and SNAP participation. Therefore, we used SNAP participation as our independent variable to measure SES, given that nearly two-thirds of the current population report an annual income less than USD $20,000 and nearly half receives SNAP assistance. Our findings illuminate a growing trend between rural residency, SNAP participation and food insecurity. It is interesting to note that SNAP participants were more likely to report being food insecure, suggesting immediate intervention is necessary within this population living in an impoverished community. Previous studies have found SNAP participation to improve food security [22]; however, our data suggest that those in SNAP are currently experiencing high rates of food insecurity. This paradox reveals high rates of reported food insecurity despite SNAP participation, leading to questions of adequate parameters or potential pitfalls. Perhaps SNAP alone is not sufficient to reduce food insecurity, yet is a necessary factor to help prevent higher rates among those who are eligible for SNAP participation. Some suggest that SNAP participants have lower FV intakes due to unobserved preferences [39] while others have found SNAP participants would be more food insecure if benefits were not available [40]. Thus, improving diet quality while simultaneously combating food insecurity requires nuance. Although SNAP participation provides an avenue to food security, it is clear that when compounded by additional factors, such as the economic disparities this rural Appalachian community experiences, it does not equate to consistent nutritional nourishment for these areas. Furthermore, rurality and geographic location are important factors to consider when it comes to food security and SNAP participation. Previous studies have even shown that rural geography contributes to differences in food perceptions and purchasing patterns, due to differences in access and availability [41,42,43]. This could explain, at least in part, the barriers in the Appalachian region.

The Appalachian region includes all or parts of 13 states where 42% of the population is rural, compared with only 20% throughout the U.S. [44]. Appalachian communities experience a high propensity for food insecurity and poverty. Across the Appalachian region, the median household income is approximately USD $48,000, compared to USD $58,000 across the U.S. [45]. Further, the central Appalachian region, which includes all of the Appalachian counties in Kentucky, maintains an annual household income of USD $36,000 [45], which may mean these communities are more disadvantaged than other rural and Appalachian communities across the U.S. Our findings further identify Martin County as representative of a geographically isolated and persistently impoverished area in Central Appalachia with exacerbated food insecurity. SNAP participation could be a mitigating factor to improve diet quality and food security among food insecure individuals [17,40,46]. Thus, food access initiatives must also consider affordability and SNAP participation with obesity prevention efforts focused on improving diet quality.

Our study also reveals that gender may influence food insecurity and would be valuable to examine in future investigations. Gender was the only covariate that had a significant effect within our sample, suggesting it may be more salient in terms of food insecurity among rural residents. The effect being stronger for females could have occurred for various reasons, including differences in other constraints for food insecurity between men and women, though we did not explore this relationship in the present study. Examining the relationship between gender and food insecurity will aid in understanding why individuals in rural communities are able or unable to access healthy foods.

Although increasing access to nutritious foods, including FV, may be challenging in a community with limited income, it is possible [47,48,49]. Rural communities continue to struggle with food accessibility within the retail food landscape, and in a particularly impoverished community, such as the one of this study, addressing the mechanisms and avenues that support food access is imperative. Recently, COVID-19 incidence has shed light on the disparities that exist between low-income and higher income households [50]. Rates of food insecurity have risen dramatically in the U.S., given a host of factors related to COVID-19 virus [51]. Thus, current programmatic and intervention efforts are critical to assist communities in dire need. Further, as Figure 1 depicts, much of the existing food system is heavily reliant on food assistance programs such as food pantries, USDA subsidized commodity foods, and small-scale church assistance [52]. Therefore, a mode of improving health status and dietary consumption would be to target these outlets and enhance offerings to individuals that utilize this network of need-based food assistance.

While environmental modifications are pivotal for sustained change, so too are policy considerations. The implications of these baseline findings align with the opportunities laid out for federal nutrition research within the U.S. [53]. This work allows us to grasp the influence of the food environment and other environmental determinants on health disparities, as well as potential relevant solutions. It is evident that policies that dictate federal nutrition program funding and allocation must consider social determinants of health within vulnerable populations. These findings also illuminate the broader systemic issues that underpin the growing rates of food insecurity within these regions exacerbated by the COVID-19 pandemic. Currently, SNAP is the only federal nutrition support offered during COVID-19, in the form of emergency allotments, adjusted verification requirements, and expanded benefits to children [54,55]. While these policy changes are temporary, they highlight larger issues and provide a platform to build upon for future research and policy development. Our findings demonstrate that providing SNAP benefits alone is not sufficient to eliminate food insecurity in rural populations. Thus, we need stronger policies that improve how we reach all vulnerable populations with improved food access.

Collectively, these findings elucidate the need for intervention, as the burden of obesity and food insecurity are major public health concerns that frequently coexist within rural communities. However, tailoring interventions to rural Appalachian communities, and other communities with unique needs such as these, is vital to implement sustainable solutions successfully. Thus, these factors should be considered together in order to construct an understanding of the intricate interrelationship between them.

In rural Appalachian communities with fewer resources, using existing assets and infrastructure are critical for community-based efforts related to food system enhancements [56]. While we are not directly influencing individual behavior with future interventions, traditional CES programing encompasses direct education focused on food procurement, preparation, preservation, and nutrition. Researchers cannot neglect these concepts when implementing community-level systems or environmental changes. Obesity prevention efforts must be multifaceted to address all levels of the socio-ecological model.

These data create a critical baseline for a prospective cohort study that will allow us to measure the ongoing work of the CDC HOP project in Martin County. Since the time of survey administration, several strategies have been implemented, or will be considered for implementation, to encourage healthy eating and active living as a well-rounded approach for obesity reduction and prevention. These strategies include nutrition and environmental audits of gas stations and grocery stores [57,58], a community garden [59], an in-store marketing initiative [60,61], a community-wide walking challenge [62,63], and enhancements to existing walking trails in partnership with the public library [64]. These approaches have led to the formation of additional partnerships within the community emphasizing healthy lifestyle behaviors. Collectively, these projects and partnerships further augment healthy eating and active living initiatives aimed at reducing obesity in this highly obese community.

### Strengths and Limitations

The generalization of the study findings is limited due to the study’s sample of participants from one rural Appalachian county in Kentucky, which may not be representative of all rural communities in Kentucky, or in other states. While verbally administered by a trained interviewer, self-reported survey responses may include social desirability or recall bias and other errors. We aimed to minimize bias by including validated survey instruments. Furthermore, researchers verbally administered the survey to reduce concerns of participants with low literacy and to reduce the likelihood of missing data due to skipping questions or not following question instructions. This format also aided in reducing survey fatigue, as the survey was 13 pages long. A portion of surveys were self-administered; however, sensitivity analyses conducted between self-administered and verbally administered surveys revealed no significant differences in findings. Results of the analyses are available upon request. Because our survey was cross-sectional, we are unable to determine causality with these results. Moreover, these are baseline data used to understand the priority population. Thus, we are not powered on interactions and results should be interpreted with caution. However, these data represent the baseline findings of a longitudinal study, which will eventually allow us to estimate causal relationships.

## 5. Conclusions

The findings of this study support continued collaboration with community networks to identify and implement strategies to address accessibility of nutritious foods. While obesity prevention efforts continue in rural areas, this community’s high obesity prevalence, coupled with low FV intakes and high food insecurity, suggests community-based efforts aimed at improving healthy food access would prove beneficial. Furthermore, tailored health-promoting initiatives that consider rurality and SNAP participation may have the greatest impact. Therefore, important next steps include leveraging existing relationships with local food providers to encourage healthier choices via food pricing and marketing strategies at their venues. Additional collaboration with local partners, such as a community health coalition and the CES, is vital to appropriately design and implement programs that consider these specific identifiers and enhance access to venues offering healthy foods.

## Figures and Tables

**Table 1:** Demographic characteristics of participants (N = 152).

Demographic Characteristic	Among All Participants % (*n*)	Among Martin County Population % ^1^
Age (mean, in years) ^2^	56 (22–84)	
Gender		
Male	34.9 (53)	55.3
Female	65.1 (99)	44.7
Race		
White	98.7 (150)	91.4
Non-white	1.3 (2)	8.5
Education		
Less than high school ^2^	43.4 (66)	
High school graduate	36.2 (55)	72.8
Post-high school	20.4 (31)	8.5
Household Income ^3^		
<USD 20,000	60.4 (90)	USD $35,000 (median)
≥USD 20,000	39.6 (59)	
Martin County Residency ^2,3^		
<20 years	17.2 (26)	
≥20 years	82.8 (125)	
SNAP Participation ^4^		
Yes	39.5 (60)	30.7
No	60.5 (92)	

^1^—Source: 2019 U.S. Census Bureau [31], ^2^—Data not available, ^3^—Numbers do not total 152 due to missing responses, ^4^—Source: SNAP Participation Map, 2019 [33].

**Table 2:** Differences in shopping practices and food security status by SNAP participation (N = 152).

Shopping Practice	SNAP			
	All % (*n*)	Participant % (*n)*	Non-Participant % (*n*)	*p*-Value
N	152	60	92	
Are you the person who usually does the grocery shopping in your household?				
Yes	70.4 (107)	71.7 (43)	69.6 (64)	0.85
No	12.5 (19)	13.3 (8)	12 (11)	
I split it with other household members	17.1 (26)	15.0 (9)	18.5 (17)	
Where do you get most of your groceries?				
Grocery store	80.3 (122)	83.3 (50)	78.3 (72)	0.44
Super center	20.4 (31)	18.3 (11)	21.7 (20)	0.61
Discount store	0.7 (1)	0.0 (0)	1.1 (1)	1.00
What is the primary reason you shop there?				
Price	42.1 (64)	50.0 (30)	37.0 (34)	0.11
Location	40.8 (62)	38.3 (23)	42.4 (39)	0.62
Quality	7.9 (12)	3.3 (2)	10.9 (10)	0.13
Variety	9.2 (14)	8.3 (5)	9.8 (9)	0.76
Are you aware of the farmers’ market in your community?				
Yes	82.9 (126)	75.0 (45)	88.0 (81)	0.05
No	17.1 (26)	25.0 (15)	12.0 (11)	
Do you regularly shop at the farmers’ market in your community?				
Yes	21.2 (32)	16.9 (10)	23.9 (22)	0.41
No	78.8 (119)	83.1 (49)	76.1 (70)	
Which of the following statements best describes the amount of food eaten in your household in the last 30 days?				
Enough food	70.9 (107)	58.3 (35)	79.1 (72)	<0.01
Not enough food	29.1 (44)	41.7 (25)	20.9 (19)	

**Table 3:** Mean FV servings by SNAP participation.

Gender	SNAP			
	Participant		Non-Participant	
	Mean FV Servings	95% CI	Mean FV Servings	95% CI
Male	2.77	(1.99–3.85)	4.45	(3.43–5.76)
Female	3.94	(3.12–4.99)	2.97	(2.45–3.60)

**Table 4:** Odds of Food Insecurity and Not Consuming Enough FV by SNAP Participation.

Characteristic	SNAP Participant vs. Non-Participant	
	Odds Ratio	95% CI
**Food Insecurity**		
Female	3.136	(1.288–7.636)
Male	2.000	(0.578–6.920)
Overall	2.707	(1.317–5.563)
**Not Enough FV**		
Female	1.366	(0.595–3.131)
Male	1.659	(0.542–5.080)
Overall	1.475	(0.761–2.859)
